# Neuropsychiatric symptoms and imbalance of atrophy in behavioral variant frontotemporal dementia

**DOI:** 10.1002/hbm.26428

**Published:** 2023-07-20

**Authors:** Andrzej Sokołowski, Ashlin R. K. Roy, Sheng‐Yang M. Goh, Emily G. Hardy, Samir Datta, Yann Cobigo, Jesse A. Brown, Salvatore Spina, Lea Grinberg, Joel Kramer, Katherine P. Rankin, William W. Seeley, Virginia E. Sturm, Howard J. Rosen, Bruce L. Miller, David C. Perry

**Affiliations:** ^1^ Department of Neurology, Memory and Aging Center, UCSF Weill Institute for Neurosciences University of California San Francisco San Francisco California USA; ^2^ Department of Pathology University of California San Francisco San Francisco California USA

**Keywords:** asymmetry, atrophy, bvFTD, dementia

## Abstract

Behavioral variant frontotemporal dementia is characterized by heterogeneous frontal, insular, and anterior temporal atrophy patterns that vary along left–right and dorso‐ventral axes. Little is known about how these structural imbalances impact clinical symptomatology. The goal of this study was to assess the frequency of frontotemporal asymmetry (right‐ or left‐lateralization) and dorsality (ventral or dorsal predominance of atrophy) and to investigate their clinical correlates. Neuropsychiatric symptoms and structural images were analyzed for 250 patients with behavioral variant frontotemporal dementia. Frontotemporal atrophy was most often symmetric while left‐lateralized (9%) and right‐lateralized (17%) atrophy were present in a minority of patients. Atrophy was more often ventral (32%) than dorsal (3%) predominant. Patients with right‐lateralized atrophy were characterized by higher severity of abnormal eating behavior and hallucinations compared to those with left‐lateralized atrophy. Subsequent analyses clarified that eating behavior was associated with right atrophy to a greater extent than a lack of left atrophy, and hallucinations were driven mainly by right atrophy. Dorsality analyses showed that anxiety, euphoria, and disinhibition correlated with ventral‐predominant atrophy. Agitation, irritability, and depression showed greater severity with a lack of regional atrophy, including in dorsal regions. Aberrant motor behavior and apathy were not explained by asymmetry or dorsality. This study provides additional insight into how anatomical heterogeneity influences the clinical presentation of patients with behavioral variant frontotemporal dementia. Behavioral symptoms can be associated not only with the presence or absence of focal atrophy, but also with right/left or dorsal/ventral imbalance of gray matter volume.

## Graphical abstract

Atrophy in behavioral variant frontotemporal dementia is heterogeneous, but most often symmetric and ventral predominant. Right/left or dorsal/ventral imbalance associates with neuropsychiatric symptom severity, with more severe eating behavior with right‐lateralized atrophy, and greater anxiety, euphoria, and disinhibition in ventral‐predominant atrophy.

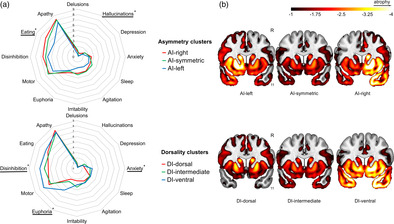

**Abbreviations**
aFTLD‐U: atypical FTLD with ubiquitin inclusions
AI: asymmetry index
bvFTD: behavioral variant frontotemporal dementia
CBD: corticobasal degeneration
CDR‐SB: Clinical Dementia Rating scale sum‐of‐boxes
DI: dorsality index
FTLD: frontotemporal lobar degeneration
MNI: Montreal Neurological Institute
NPI: Neuropsychiatric Inventory

## 1 INTRODUCTION

Behavioral variant frontotemporal dementia (bvFTD) is a neurodegenerative syndrome characterized by changes in emotion, social function, and personality, with characteristic atrophy of frontal, temporal, and insular cortices, as well as degeneration of subcortical regions, including the striatum and amygdala (Pan et al., 2012; Seeley et al., 2008). While there is substantial overlap in the atrophy patterns of patients with bvFTD (Perry et al., 2017), there is also heterogeneity in terms of asymmetry or the degree of dorsal or ventral degeneration (Fukui & Kertesz, 2000; Ranasinghe et al., 2016; Seeley et al., 2008; Whitwell, Przybelski, et al., 2009; Whitwell et al., 2013).

Although descriptions of atrophy in patients with bvFTD sometimes emphasize greater right hemisphere degeneration (Fukui & Kertesz, 2000; Seeley et al., 2008), other studies indicate that atrophy is more often symmetric and less commonly right‐ or left‐predominant (Whitwell et al., 2013). Little is known about how the degree of structural asymmetry affects bvFTD clinical symptomatology. Brain asymmetry has been linked to numerous cognitive and behavioral functions. The left hemisphere is typically dominant for language. Models of socioemotional function either assert a primary role of the right hemisphere or propose hemispheric lateralization; for example, left hemisphere control of positive emotion or approach behavior and right hemisphere predominance for negative emotion or withdrawal (Alves et al., 2008; Davidson, 1992; Gainotti, 2019). There have been prior investigations of the relationship between structural asymmetry and behavioral profile in patients with bvFTD (Carr et al., 2020; Gainotti, 2019; Irwin et al., 2018; Liu et al., 2004; Miller et al., 1993; Mychack et al., 2001; Sturm et al., 2015; Whitwell et al., 2013), but this matter has not been fully explored. Greater severity of core bvFTD symptoms has often been associated with right hemisphere degeneration (Gainotti, 2019; Irwin et al., 2018; Liu et al., 2004; Miller et al., 1993; Mychack et al., 2001). One study compared patients with symmetric and asymmetric atrophy patterns in terms of overall neuropsychiatric symptoms and found no differences (Whitwell et al., 2013). Fewer studies have associated behavioral features in bvFTD with left‐lateralized lesions, though increased positive emotional reactivity has been described with left frontal atrophy (Sturm et al., 2015). Frontal asymmetry has been related to a higher agitation and irritability (Carr et al., 2020), although this study did not investigate the direction of asymmetry.

In addition to asymmetry, the dorsal or ventral predominance of atrophy can differ among patients with bvFTD and could also relate to symptom heterogeneity. Dorsal prefrontal regions are responsible for cognitive and behavioral control as well as emotion regulation. Conversely, ventral frontotemporal regions and ventral subcortical structures, such as nucleus accumbens and amygdala are involved in salience detection, reward, and emotional reactivity (Etkin et al., 2015; Rosen et al., 2005). Data‐driven approaches have grouped patients with bvFTD into distinct patterns of atrophy, with some groups involving more frontal atrophy, some more temporal, or others involving both equally (Ranasinghe et al., 2016; Whitwell, Przybelski, et al., 2009). These studies have differed in their determination of whether these atrophy patterns are associated with distinct behavioral profiles. Most patients with bvFTD have an underlying pathological diagnosis of frontotemporal lobar degeneration (FTLD). FTLD is divided into subtypes, with some associated with dorsal or ventral predominance of atrophy. In a prior autopsy study, we found that subtypes that on average show greater temporal lobe involvement displayed greater loss of empathy and compulsive behavior than subtypes that tended to involve more dorsal frontal atrophy (Perry et al., 2017). Temporal lobe involvement, particularly on the right side, can occur to a variable extent in patients with ventral atrophy in bvFTD and has been associated with a variety of behavioral features (Chan et al., 2009; Rosso et al., 2001; Thompson et al., 2003). To our knowledge, previous studies have not specifically investigated clinical profiles associated with an imbalance between dorsal and ventral atrophy, referred to here as “dorsality.”

The goal of this study was to investigate the frequency and clinical correlates of frontotemporal asymmetry (right vs. left) and dorsality (ventral vs. dorsal predominance) of atrophy in a large cohort of patients with bvFTD. We aimed to describe the severity of neuropsychiatric symptoms in patients who differ in imbalance of brain atrophy. We hypothesized that bvFTD patients with right‐lateralized frontotemporal atrophy would exhibit greater disinhibition and abnormal eating behaviors whereas those with left‐lateralized atrophy would show greater speech and language impairments (Gainotti, 2019; Perry et al., 2017; Regard & Landis, 1997; Whitwell et al., 2007; Woolley et al., 2007). We also hypothesized that there would be greater cognitive and executive impairment in patients with dorsal predominant atrophy and more prominent socioemotional symptoms in the ventral predominant atrophy group.

## 2 MATERIALS AND METHODS

### 2.1 Participants

All participants underwent a comprehensive evaluation at the University of California San Francisco (UCSF). We identified patients at the UCSF Memory and Aging Center who were given a bvFTD clinical diagnosis, met International Frontotemporal Dementia Criteria Consortium (FTDC) criteria for at least possible bvFTD (Rascovsky et al., 2011), underwent a structural MRI scan, and had a neuropsychiatric assessment. A total of 250 patients with bvFTD (99 females) aged 29–83 (*M* = 61.43; *SD* = 8.82) took part in the study. Participants underwent a neuropsychological assessment that included tests of memory, visuospatial abilities, language, and executive function (Kramer et al., 2003). The Clinical Dementia Rating scale sum‐of‐boxes (CDR‐SB) (Morris, 1993) was used to measure the severity of global functional impairment. All assessments were conducted within 6 months of the MRI scans. Written informed consent was obtained from patients or surrogates according to procedures approved by the UCSF Committee on Human Research.

### 2.2 Neuropsychiatric assessment

Behavioral impairment was assessed via subscale scores (frequency × severity), rated by an informant for each of the 12 domains from the Neuropsychiatric Inventory (NPI) (Cummings et al., 1994). The Interpersonal Reactivity Index (Davis, 1983) was used to measure empathy. The assessment was conducted within 6 months from MRI scans.

### 2.3 Image acquisition

Whole‐brain T1‐weighted images were acquired on two sites using four MRI scanners: (a) 3 T MRI Trio Tim (*n* = 95) and (b) 3 T MRI Prisma Fit (*n* = 79) scanners at the UCSF Neuroscience Imaging Center; (c) 1.5 T Magnetom Vision system (*n* = 56); and (d) 4 T Bruker MedSpec with Trio console (*n* = 20) scanners at the San Francisco VA Medical Center. The acquisition parameters have been previously published (Bettcher et al., 2012; La Joie et al., 2021; Mueller et al., 2009; Rosen et al., 2002).

### 2.4 Genetics and pathological diagnosis

To assess whether asymmetry or dorsality differed based on mutation status or pathological diagnosis, we obtained genetic and pathological results whenever available. Patients were screened using available blood or frozen tissue samples for genetic mutations known to cause autosomal dominant inheritance of FTD or Alzheimer's disease (*APP, C9orf72*, *FUS, GRN*, *MAPT, PSEN1*, *PSEN2*, and *TARDBP*). Data were available for 240 patients.

Postmortem neuropathological assessment followed previously described procedures (Forman et al., 2006; Tartaglia et al., 2010). Consensus diagnostic criteria were used to establish pathological diagnoses (Cairns et al., 2007; Hyman et al., 2012; Hyman & Trojanowski, 1997; MacKenzie et al., 2010; Mackenzie et al., 2011). A pathological diagnosis was available for 95 patients.

### 2.5 Atrophy maps

All structural images were visually inspected for the presence of excessive motion artifacts. Preprocessing included segmentation into multiple brain tissues, alignment and normalization to the standard adult tissue probability maps templates in Montreal Neurological Institute (MNI) space distributed with SPM12 (http://www.fil.ion.ucl.ac.uk/spm/), and modulation and smoothing with an 8 mm full width at half maximum Gaussian kernel.

Voxel‐wise atrophy statistical maps were estimated using W‐scores in reference to a healthy control group (La Joie et al., 2012; Perry et al., 2017). Each structural image underwent a visual quality check, and the transformation to the template space was also checked for quality. To assess the accuracy of the fit, the r‐squared coefficient of determination at each voxel was utilized. The use of W‐scores prevents the attribution of normal differences or those related to covariates to disease or behaviors of interest. Multiple linear regression, on the reference group, was run in each voxel as a function of age, scanner type, and total intracranial volume. The healthy control group was regressed using a sample of 383 cognitively normal controls assessed at the UCSF Memory and Aging Center. Patient W‐scores are equal to the difference between measured and expected gray matter volume divided by the standard deviation in controls ([actual − expected]/SD). The expected and the SD for the patients were estimated using the parameters fitted with the group of controls. W‐scores have a mean value of 0 and a standard deviation of 1; scores below 0 represents low volume relative to controls.

### 2.6 Frontotemporal mask

**FIGURE 1:**
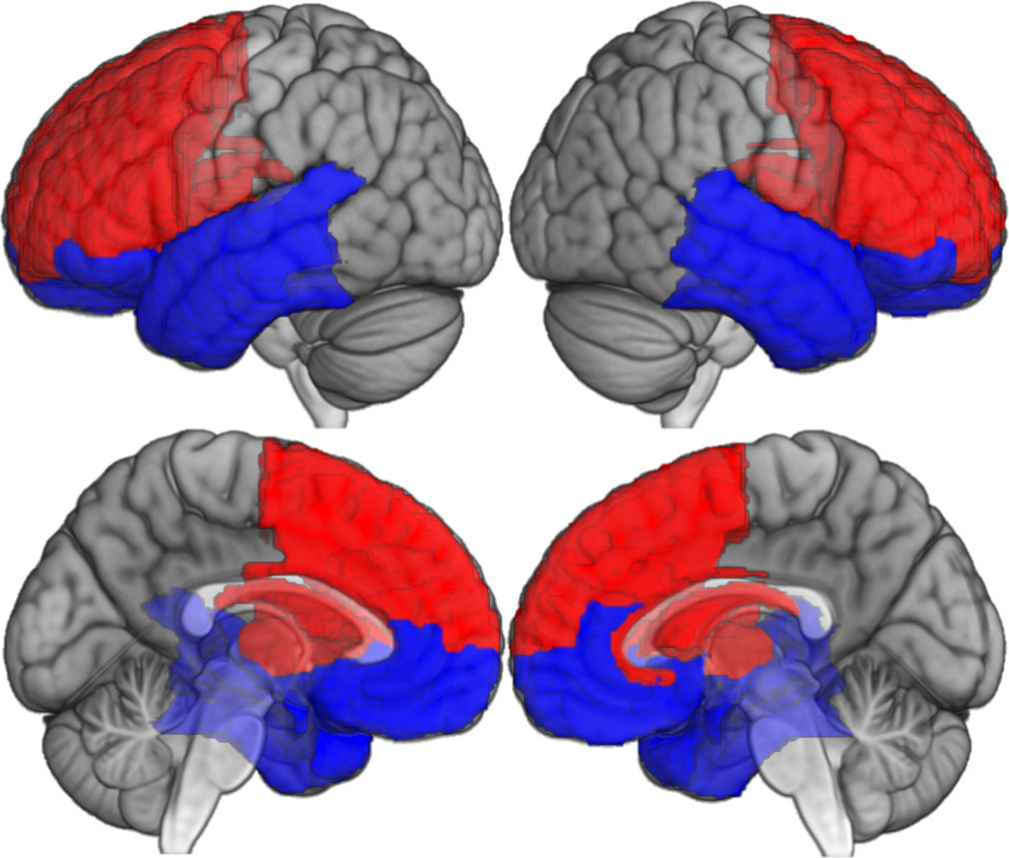
Frontotemporal regions of interest. Ventral regions are presented in blue, dorsal regions are presented in red.

We generated a frontotemporal mask (Figure 1) by extracting W‐scores from areas that frequently show atrophy in bvFTD, using regions of interest taken from the Brainnetome atlas (Fan et al., 2016), including regions in the prefrontal and temporal cortices, insula, and striatum (nucleus accumbens and dorsal caudate and putamen). Importantly, subcortical structures and insula were divided into ventral (ventral insula, amygdala, nucleus accumbens) and dorsal (dorsal insula, dorsal caudate, and dorsolateral putamen) regions. We divided this mask into ventral and dorsal regions separately for left and right hemisphere, forming frontotemporal quadrants. Full list of regions is reported in supplementary material.

### 2.7 Asymmetry and dorsality indices

An asymmetry index (AI) was calculated as a difference between mean W‐scores for all voxels in the right and left frontotemporal regions. Positive AI represents left‐lateralized frontotemporal atrophy (greater atrophy on left than right) and negative AI represents right‐lateralized frontotemporal atrophy. A dorsality index (DI) was calculated as a difference between the W‐scores in dorsal and ventral frontotemporal regions. Positive DI represents ventral‐predominant frontotemporal atrophy and negative DI represents dorsal‐predominant frontotemporal atrophy. AI and DI indices were used as continuous variables. To determine the effect of right versus left or dorsal versus ventral imbalances within specific quadrants we generated values for right DI and left DI (dorsal minus ventral mean W‐scores only in right or left hemispheric regions), as well as dorsal AI and ventral AI (right minus left mean W‐scores only in dorsal or ventral regions).

### 2.8 Behavioral analysis

#### 2.8.1 Cluster analysis

The frequency of asymmetric frontotemporal atrophy as well as predominance of ventral or dorsal frontotemporal atrophy was determined using AI and DI. Each of the two indices was separately entered into a cluster analysis using k‐means clustering. A three‐cluster solution was specified with the intent to identify left/symmetric/right and ventral/intermediate/dorsal atrophied groups. The clustering method was applied to provide an objective criterion for comparative group assignment and to allow for imbalance in group size depending on the distribution of the data. ANCOVA was used to test group differences in NPI and neuropsychological functioning between clusters. Post hoc pairwise comparisons with Bonferroni correction were performed. Since age, scanner type, and total intracranial volume were already accounted for upon calculating W‐scores, analyses were additionally controlled for sex and CDR‐SB. All behavioral analyses were performed in IBM SPSS v.27.

#### 2.8.2 Genetic and neuropathology analysis

Differences in NPI scores between carriers of different gene mutations (and those who tested negative for all mutations) and between patients with different neuropathological diagnoses were measured by separate ANCOVAs controlling for sex. The differences in frequency of each gene mutation and neuropathological diagnosis based on asymmetry and dorsality clustering were measured using *χ* ^2^ tests. Groups with at least four patients were entered into the analyses.

### 2.9 Imaging correlates of symptom severity

Stepwise regression analyses were performed to determine if neuropsychiatric symptoms were predicted by the frontotemporal atrophy, AI, and DI in the entire sample. W‐scores from the frontotemporal quadrants, dorsal and ventral AI, as well as right and left DI were entered as predictors of NPI scores. The effects of age, scanner, and total intracranial volume having been accounted for in the calculation of W maps, only sex was entered as a nuisance variable.

To further explore the relationship between neuropsychiatric symptom severity and whole‐brain gray matter volume voxel‐wise regressions were performed with NPI scores as predictors of W‐scores separately for each behavior. Analyses were performed in SPM12. Results are reported at a peak threshold of *p* < .001, with cluster‐correction threshold of FWE *p* < .05 and displayed at the uncorrected threshold of *p* < .01 for illustrative purposes. Coordinates are reported in MNI space.

### 2.10 Additional investigations of asymmetry and dorsality

We further investigated behaviors that were significantly related to either asymmetry or dorsality in the cluster analysis. The goal of these investigations was to distinguish between distinct potential interpretations of a significant relationship between AI or DI and a behavior. For example, a correlation between AI and a behavior could relate to strong unilateral atrophy or preservation, to independent and opposite effects of atrophy or preservation in each hemisphere, or to an interaction between volume in one hemisphere with volume in the other. First, to investigate whether there was an interaction effect, we entered the main effects of left and right frontotemporal atrophy and their interaction or dorsal and ventral frontotemporal atrophy and their interaction as predictors of neuropsychiatric symptoms into multiple regression models predicting NPI scores. Sex was entered as a nuisance variable. Additionally, we plotted the interactions based on asymmetry or dorsality for visual assessment.

Second, for the behaviors that were associated with asymmetry in the cluster analysis, we performed additional voxel‐wise analyses to investigate whether cluster‐level asymmetry findings were independently associated with voxel‐level asymmetry and whether voxel‐level asymmetry was solely driven by volume in each hemisphere or by an interaction between right‐ and left‐sided volume. The voxel‐wise asymmetry analysis calculated the degree of asymmetry as the difference between right and left hemisphere volume at each voxel. Analyses involving hemispheric atrophy and their interaction were performed using Biological Parametric Mapping toolbox (BPM 3.1) (Casanova et al., 2007) in SPM5 (BPM is not supported in recent SPM releases). The toolbox permits the inclusion of voxel‐wise maps as imaging covariates. Three maps were entered into each of these analysis—one for ipsilateral atrophy, one for contralateral atrophy (derived by flipping the image on the left‐to‐right axis), and one for the left × right interaction. The interaction map was calculated as a multiplication of the ipsilateral and contralateral W maps. Due to the symmetric nature of these maps (volume in right voxels accounting for volume of the flipped left voxel or left volume accounting for flipped right), all analyses were limited to the right hemisphere. There were two voxel‐wise regressions for each behavior. One assessed the ability of atrophy to predict the NPI score when controlling for contralateral atrophy and the ipsilateral × contralateral interaction. The other assessed the ability of the interaction to predict behavior when correcting for ipsilateral and contralateral W score. All analyses were controlled for sex. As with other voxel‐wise atrophy analyses, results were displayed at the uncorrected threshold of *p* < .01, with significance set at peak *p* < .001; FWE *p* < .05 cluster corrected.

## 3 RESULTS

### 3.1 Behavioral analysis

A total of 250 patients with bvFTD (99 females) aged 29–83 (*M* = 61.43; *SD* = 8.82) were investigated. Their CDR‐SB scores reflect that most patients fall in a mild dementia level of severity. The most common symptoms across the sample were apathy (*M* = 7.97; *SD* = 3.63), eating behavior (*M* = 6.24; *SD* = 4.01), aberrant motor behavior (*M* = 6.12; *SD* = 4.37), and disinhibition (*M* = 6.08; *SD* = 3.83).

The extent of atrophy was examined in patients across diagnoses. The mean frontotemporal W scores were *M* = −0.77; *SD* = 0.72 for possible bvFTD according to FTDC (*n* = 22), *M* = −1.15; *SD* = 0.60 for probable bvFTD (*n* = 120), *M* = −1.15; *SD* = 0.57 for definite bvFTD (*n* = 61), and *M* = −1.21; *SD* = 0.67 among those meeting Neary criteria (*n* = 47).

**FIGURE 2:**
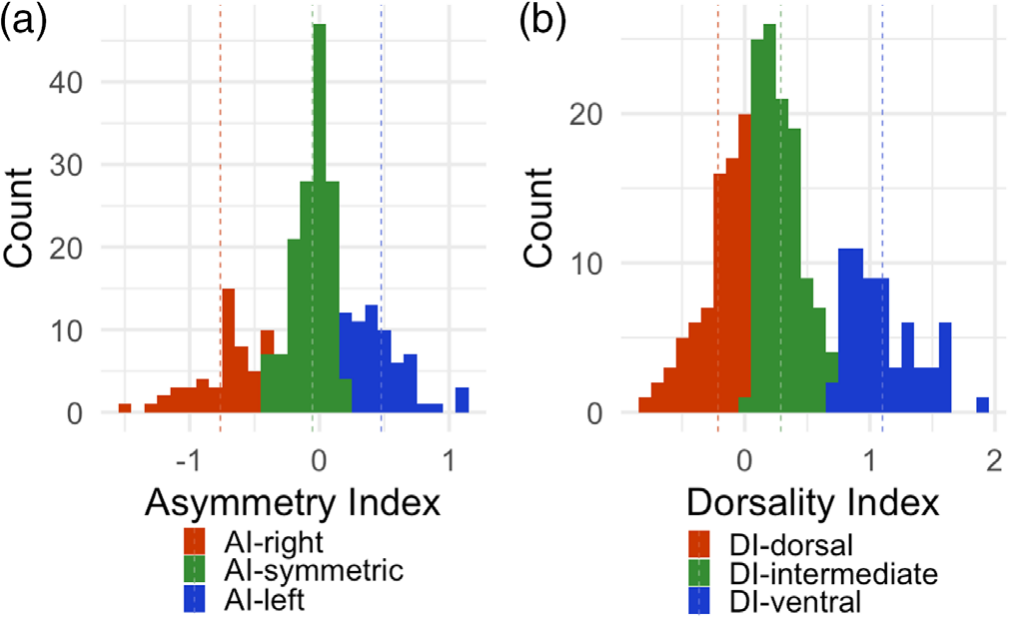
The frequency of frontotemporal asymmetry (a) and predominance of ventral or dorsal frontotemporal atrophy (b), with groups derived by k‐means clustering.

Across the sample, neither AI nor DI were normally distributed using the Kolmogorov–Smirnov test, *D*(250) = .11 and .10, respectively; *p* < .001. The distribution of AI was fairly balanced (*M* = −.06; *SD* = .03; skewness −0.39; kurtosis = 0.55). Then, 42 (17%) patients had AI below −0.5 *SD* and 23 (9%) had AI above 0.5 *SD* suggesting relatively similar frequency of right‐ and left‐sided lateralization (respectively). The distribution of DI was skewed towards ventral predominant atrophy (*M* = .34; *SD* = .03; skewness = 0.56; kurtosis = −0.20 (see Figure 2)). Seven (3%) patients had DI below −0.5 *SD* and 79 (32%) were above 0.5 *SD* indicating more frequent ventral predominant atrophy.

### 3.2 Cluster analysis

Cluster analysis provided a three‐cluster solution based on each index (Figure 2). There were three clusters derived from AI: right‐lateralized (AI‐right; *n* = 48; *M* = −0.76; *SD* = 0.24), symmetric (AI‐symmetric; *n* = 142; *M* = −0.05; *SD* = 0.14), and left‐lateralized (AI‐left; *n* = 60; *M* = 0.48; *SD* = 0.21). K means defined three DI clusters: dorsal predominant (DI‐dorsal; *n* = 76; *M* = −0.21; *SD* = 0.20), without dorsal or ventral predominance (DI‐intermediate; *n* = 110; *M* = 0.29; *SD* = 0.17), and ventral predominant (DI‐ventral; *n* = 64; *M* = 1.10; *SD* = 0.28).

**FIGURE 3:**
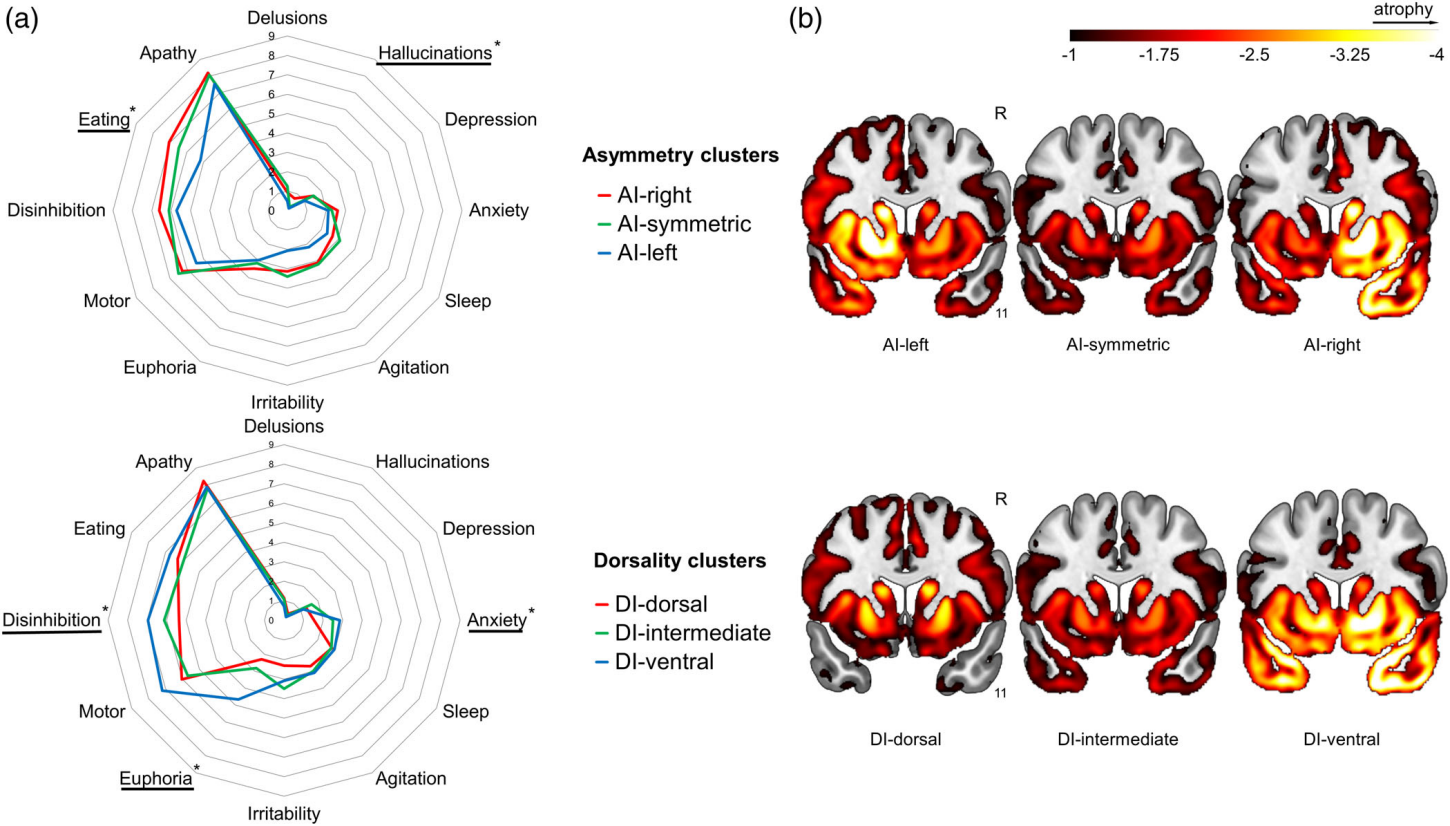
(a) Mean NPI scores in groups based on AI and DI. (b) Mean gray matter atrophy for each group represented by W‐scores < −1. AI, asymmetry index; DI, dorsality index; * significant group differences.

While there were many similarities, there were also notable differences in the cognitive and behavioral profiles among the AI‐ and DI‐based clusters (Table 1 and Figure 3). Asymmetry clusters differed in hallucinations (*F* = 3.32; *p* < .05) and eating behavior (*F* = 3.42; *p* < .05) scores, driven by higher scores in the AI‐right cluster. Patients in the AI‐left cluster had greater impairment on cognitive tests involving language (naming, verbal fluency), memory, and certain aspects of attention or executive function. Dorsality clusters differed in euphoria (*F* = 8.25; *p* < .001), anxiety (*F* = 3.3; *p* < .05), and disinhibition (*F* = 2.95; *p* < .05) scores, with higher scores in the DI‐ventral group. Patients in the DI‐ventral cluster generally had less difficulty with tests of executive function and working memory than clusters that involved a greater proportion of dorsal atrophy; however, those in the DI‐ventral cluster had more difficulty with certain tasks that could reflect greater temporal lobe involvement (naming and word meaning as well as visual memory).

**TABLE 1:** Group differences in neuropsychiatric symptoms and neuropsychological assessment.

		AI clusters			DI clusters		
	All cases	AI‐right	AI‐symmetric	AI‐left	DI‐dorsal	DI‐intermediate	DI‐ventral
	*n* = 250	*n* = 48	*n* = 142	*n* = 60	*n* = 76	*n* = 110	*n* = 64
CDR‐SB	6.98 (3.46)	6.60 (3.17)	6.88 (3.40)	7.52 (3.79)	6.96 (3.44)	7.19 (3.70)	6.66 (3.09)
NPI Ag	2.92 (3.42)	3.08 (3.85)	3.17 (3.41)	2.22 (3.02)	2.66 (3.30)	3.00 (3.41)	3.09 (3.62)
NPI Anx	2.33 (3.24)	2.73 (3.85)	2.28 (3.23)	2.13 (2.72)	**1.54** ^**a**^ **(2.47)**	**2.57** ^**a,b**^ **(3.23)**	**2.86** ^**b**^ **(3.87)**
NPI Apth	7.97 (3.63)	8.19 (3.15)	8.07 (3.79)	7.53 (3.65)	8.23 (2.99)	7.83 (3.99)	7.89 (3.74)
NPI Del	1.03 (2.41)	0.96 (2.38)	1.29 (2.70)	0.47 (1.47)	1.18 (2.56)	1.10 (2.51)	0.72 (2.04)
NPI Dep	1.41 (2.61)	1.50 (2.66)	1.55 (2.61)	1.00 (2.55)	1.29 (2.43)	1.65 (2.72)	1.14 (2.61)
NPI Dis	6.08 (3.83)	6.60 (3.39)	6.06 (4.12)	5.69 (3.47)	**5.34** ^**a**^ **(3.78)**	**6.07** ^**a,b**^ **(3.86)**	**6.95** ^**b**^ **(3.73)**
NPI Eat	6.24 (4.01)	**6.89** ^**a**^ **(3.26)**	**6.48** ^**b**^ **(4.18)**	**5.20** ^**b**^ **(4.01)**	6.28 (4.14)	5.93 (4.18)	6.73 (3.55)
NPI Eup	3.11 (3.68)	3.38 (3.50)	3.11 (3.81)	2.90 (3.56)	**2.26** ^**a**^ **(3.25)**	**2.78** ^**a**^ **(3.59)**	**4.67** ^**b**^ **(3.90)**
NPI Hal	0.29 (1.29)	**0.71** ^**a**^ **(2.41)**	**0.22** ^**a,b**^ **(0.83)**	**0.13** ^**b**^ **(0.79)**	0.38 (1.13)	0.28 (1.27)	0.20 (1.50)
NPI Irr	3.00 (3.90)	3.15 (4.05)	3.37 (4.20)	2.03 (2.79)	2.28 (3.27)	3.45 (4.14)	3.09 (4.09)
NPI Mot	6.12 (4.37)	6.15 (4.56)	6.40 (4.35)	5.44 (4.28)	5.96 (4.59)	5.59 (4.25)	7.19 (4.19)
NPI Sle	2.85 (3.70)	2.62 (3.39)	3.14 (4.01)	2.36 (3.14)	2.947 (3.46)	2.72 (3.80)	2.97 (3.86)
CVLT 10 min	3.2 (2.80)	**4.07** ^**a**^ **(2.55)**	**3.58** ^**a,b**^ **(2.94)**	**1.65** ^**b**^ **(1.99)**	3.16 (2.75)	3.53 (2.74)	2.66 (2.94)
Modified trails B time	70.68 (39.02)	**83.03** ^**a**^ **(39.30)**	**64.24** ^**b**^ **(38.11)**	**74.52** ^**a,b**^ **(38.54)**	**84.55** ^**a**^ **(40.03)**	**68.51** ^**a,b**^ **(38.00)**	**58.56** ^**b**^ **(35.19)**
Modified trails B errors	1.96 (2.39)	2.03 (2.46)	2.04 (2.56)	1.72 (1.89)	**2.65** ^**a**^ **(2.33)**	**1.93** ^**a,b**^ **(2.53)**	**1.21** ^**b**^ **(2.02)**
Design fluency	5.53 (3.77)	5.67 (3.90)	5.71 (3.73)	5.06 (3.81)	4.72 (3.50)	5.77 (3.72)	6.19 (4.06)
Benson figure copy	14.13 (2.89)	14.93 (1.84)	13.95 (2.90)	13.96 (3.40)	**13.54** ^**a**^ **(3.08)**	**13.82** ^**a**^ **(3.23)**	**15.45** ^**b**^ **(1.03)**
Benson figure delayed recall	6.82 (4.38)	**6.07** ^**a,b**^ **(4.20)**	**7.65** ^**a**^ **(4.34)**	**5.49** ^**b**^ **(4.24)**	**7.89** ^**a**^ **(4.41)**	**7.06** ^**a**^ **(3.90)**	**5.05** ^**b**^ **(4.65)**
Digits forward	5.58 (1.42)	**6.24** ^**a**^ **(1.38)**	**5.52** ^**b**^ **(1.32)**	**5.28** ^**b**^ **(1.55)**	**5.61** ^**a,b**^ **(1.27)**	**5.28** ^**a**^ **(1.45)**	**6.07** ^**b**^ **(1.44)**
Digits backward	3.65 (1.61)	**4.51** ^**a**^ **(1.34)**	**3.63** ^**b**^ **(1.54)**	**3.07** ^**b**^ **(1.69)**	**3.11** ^**a**^ **(1.57)**	**3.51** ^**a**^ **(1.51)**	**4.55** ^**b**^ **(1.48)**
D‐word fluency	6.99 (5.06)	**7.88** ^**a**^ **(3.73)**	**7.63** ^**a**^ **(5.49)**	**4.79** ^**b**^ **(4.24)**	**5.16** ^**a**^ **(4.71)**	**7.63** ^**b**^ **(5.12)**	**8.23** ^**b**^ **(4.79)**
Animal fluency	10.61 (6.64)	**11.07** ^**a,b**^ **(5.44)**	**11.54** ^**a**^ **(6.67)**	**8.00** ^**b**^ **(6.87)**	**8.12** ^**a**^ **(6.22)**	**12.00** ^**b**^ **(6.53)**	**11.35** ^**b**^ **(6.58)**
BNT correct	11.17 (3.81)	**11.24** ^**a,b**^ **(4.28)**	**11.77** ^**a**^ **(3.41)**	**9.76** ^**b**^ **(4.00)**	**12.33** ^**a**^ **(2.70)**	**11.75** ^**a**^ **(3.38)**	**8.73** ^**b**^ **(4.58)**
PPVT‐R	12.81 (3.54)	13.00 (3.52)	13.15 (3.45)	11.88 (3.65)	**12.88** ^**a,b**^ **(3.59)**	**13.49** ^**a**^ **(2.96)**	**11.64** ^**b**^ **(4.04)**
Stroop CNC	58.48 (24.26)	**66.37** ^**a**^ **(26.63)**	**59.89** ^**a**^ **(22.55)**	**48.94** ^**b**^ **(24.34)**	**48.09** ^**a**^ **(25.66)**	**59.09** ^**a,b**^ **(21.45)**	**68.65** ^**b**^ **(22.70)**
Stroop IC	32.5 (18.14)	**37.77** ^**a**^ **(18.01)**	**33.45** ^**a**^ **(18.47)**	**25.92** ^**b**^ **(15.81)**	**23.91** ^**a**^ **(16.52)**	**31.27** ^**a**^ **(16.87)**	**42.96** ^**b**^ **(16.68)**
CATS‐FM	10.53 (1.88)	10.42 (2.36)	10.49 (1.77)	10.68 (1.83)	**9.90** ^**a**^ **(2.21)**	**10.61** ^**a,b**^ **(1.79)**	**11.12** ^**a**^ **(1.33)**
CATS‐AM	9.33 (3.18)	10.00 (2.43)	9.44 (3.26)	8.63 (3.32)	**7.96** ^**a**^ **(3.07)**	**10.26** ^**b**^ **(2.64)**	**9.37** ^**a,b**^ **(3.62)**
GDS	7.98 (6.64)	7.69 (6.29)	7.90 (6.58)	8.40 (7.18)	9.46 (6.23)	7.44 (6.44)	7.06 (7.24)
IRI EC	18.95 (6.86)	20.2 (6.13)	18.67 (6.99)	18.71 (7.09)	**18.6** ^**a,b**^ **(6.14)**	**20.06** ^**a**^ **(7.19)**	**17.4** ^**b**^ **(6.96)**
IRI PT	15.02 (5.26)	16.63 (5.96)	14.74 (5.37)	14.52 (4.37)	**14.34** ^**a,b**^ **(4.52)**	**16.1** ^**a**^ **(5.48)**	**14.22** ^**b**^ **(5.73)**
IRI FS	20.32 (5.93)	21.21 (6.20)	20.22 (6.33)	19.95 (4.78)	19.65 (5.92)	21.22 (5.90)	19.8 (5.94)
IRI PD	13.31 (5.37)	13.59 (5.28)	12.84 (5.39)	14.21 (5.38)	12.92 (4.74)	14.3 (5.81)	11.98 (5.06)

*Note*: Group differences between AI clusters and between DI clusters are reported using superscript notation. The values with different superscript letters in a row are significantly different (*p* < .05). Means sharing the same superscript are not significantly different from each other. Models were tested separately for AI and DI groups. Means are reported with standard deviations in the brackets. Bold values reflect a significat omnibus ANCOVA between the three clusters, with superscript notation indicating the results of posthoc pairwise comparisons.

Abbreviations: AI, asymmetry index; BNT, Boston Naming Test; CATS, Comprehensive Affect Testing System; CATS‐AM, CATS Affect Matching; CATS‐FM, CATS Facial Matching; CDR‐SB, Clinical Dementia Rating scale sum of boxes; CVLT, California Verbal Learning Test; DI, Dorsality Index; GDS, Geriatric Depression Scale; IRI, interpersonal reactivity index; IRI EC, IRI empathic concern; IRI FS, IRI fantasy; IRI PD, IRI personal distress; IRI PT, IRI perspective‐taking; NPI, Neuropsychiatric Inventory; NPI Ag, Agitation; NPI Anx, Anxiety; NPI Apth, Apathy; NPI Del, Delusions; NPI Dep, Depression; NPI Dis, Disinhibition; NPI Eat; Eating behavior; NPI Eup; Euphoria; NPI Hal, Hallucinations; NPI Irr, Irritability; NPI Mot; Aberrant Motor Behavior; NPI Sle; Sleep changes; PPVT‐R, Peabody Picture Vocabulary Test—Revised; Stroop CNC, Color Naming Correct; Stroop IC, interference correct.

### 3.3 Atrophy clusters and behavior by genetic status

There was a difference in the frequency of the *C9orf72* repeat expansion between both asymmetry and dorsality groups. The *C9orf72* repeat expansion was more common in the AI‐symmetric group (29% of those tested in a group) than both AI‐right and AI‐left groups (0 and 7%, respectively; *χ* ^2^ = 25.97; *p* < .001). It was also more common in DI‐intermediate group (31%) than in both DI‐dorsal and DI‐ventral groups (14 and 2%, respectively; *χ* ^2^ = 24.12; *p* < .001). *MAPT* mutations were present more often in the DI‐ventral group (15%) than both DI‐dorsal and DI‐intermediate groups (0 and 3%, respectively; *χ* ^2^ = 16.54; *p* < .001). *GRN* mutations were less often present in the AI‐symmetric (4%) than in the AI‐right and AI‐left (7 and 11%, respectively) groups, although the group differences did not reach statistical significance. The frequencies of genetic mutations are reported in Supplementary Table 1.

Carriers of different mutations differed in NPI euphoria scores (*F*(3,223) = 3.24; *p* = .023), with *MAPT* carriers (*M* = 5.25; *SD* = 4.77) displaying higher mean scores than *C9orf72* carriers (*M* = 1.88; *SD* = 3.21). Groups also differed in NPI eating behavior (*F*(3,221) = 4.09; *p* = .007), with patients who tested negative for genetic mutations (*M* = 6.97; *SD* = 3.89) having significantly higher scores than those with *C9orf72* expansions (*M* = 4.67; *SD* = 4.17). Group statistics are reported in Supplementary Table 2.

### 3.4 Atrophy clusters and behavior by pathological diagnosis

The frequency of some neuropathological diagnoses differed among clusters. Pick's disease was more common in the AI‐right group (68% of those in that group who came to autopsy) than in both AI‐left and AI‐symmetric groups (5 and 0%, respectively; *χ* ^2^ (2, *N* = 95) = 54.12; *p* < .001). TDP‐B was more common in the AI‐symmetric group (35%) than in the AI‐right group (0%; *χ* ^2^ (2, *N* = 95) = 10.67; *p* = .005). TDP‐C was more common in the AI‐right group (14%) than in the AI‐symmetric groups (0%; *χ* ^2^ (2, *N* = 95) = 7.15; *p* = .028), reflecting the inclusion of patients with right temporal bvFTD. The frequency of neuropathological diagnoses also differed among dorsality groups. Corticobasal degeneration (CBD) was more common in the DI‐dorsal group (24%) than in DI‐ventral and DI‐intermediate groups (0 and 3%, respectively; *χ* ^2^ (2, *N* = 95) = 12.36; *p* = .002). TDP‐A was also more common in the DI‐dorsal group (30%) than in the DI‐ventral group (0%; *χ* ^2^ (2, *N* = 95) = 10.04; *p* < .001). On the other hand, TDP‐C was more common in the DI‐ventral group (19%) than in DI‐dorsal and DI‐intermediate groups (both 0%; *χ* ^2^ (2, *N* = 95) = 14.72; *p* = .001). The frequencies of neuropathological diagnoses are reported in Supplementary Tables 3 and 4.

Patients with different neuropathological diagnoses differed in NPI agitation scores, *F*(6,73) = 2.72; *p* = .019, such that patients with atypical FTLD with ubiquitin inclusions (aFTLD‐U) had higher scores than those with CBD and TDP‐A. There were also differences in NPI euphoria scores, *F*(6,71) = 3.76; *p* = .003. Scores among those with aFTLD‐U were significantly higher than those with Pick's disease, TDP‐A, TDP‐B, or TDP‐unclassifiable. Patients also differed in NPI Sleep scores (*F*(6,71) = 3.47; *p* = .005). Patients with aFTLD‐U had higher scores than CBD, TDP‐A, TDP‐B, TDP‐C, and TDP‐unclassifiable. Group statistics are reported in Supplementary Table 5.

### 3.5 Imaging correlates of symptom severity

Stepwise regression models using volume in four frontotemporal quadrants, dorsal and ventral AI, and right and left DI yielded significant volumetric predictors of neuropsychiatric symptom severity, informing whether each behavior was primarily driven by high or low regional gray matter volume, or whether right/left or dorsal/ventral imbalance was a better predictor. Dorsality findings were significant predictors of all three behaviors that differed among DI‐based clusters. Anxiety was predicted by (1) high right dorsal W‐scores (*β* = .14; *t*(245) = 2.26; *p* = .025), suggesting relative preservation of dorsal volume, and (2) positively by right DI (*β* = .14; *t*(245) = 2.3; *p* = .022; overall model—*F*(3,245) = 4.21; *p* = .006), indicating ventral predominance of atrophy in the right hemisphere. Disinhibition was positively predicted by left DI (*β* = .21; *t*(242) = 3.37; *p* = .001; overall model—*F*(2,242) = 5.81; *p* = .003). Euphoria was also positively predicted by left DI (*β* = .24; *t*(244) = 3.94; *p* < .001; overall model—*F*(2,244) = 8.24; *p* < .001). Among behaviors that differed based on AI‐based clusters, eating behavior was negatively predicted by dorsal AI (*β* = −.183; *t*(242); *p* = .004; overall model—*F*(2,242) = 5.07; *p* = .007), reflecting right lateralization; however, Hallucinations were predicted by right dorsal atrophy (*β* = −.175; *t*(247) = −2.81; *p* = .005; overall model—*F*(2,247) = 4.44; *p* = .013), not by a measure of AI. Other behaviors were best predicted by high W‐scores, indicating a relative preservation of regional gray matter volume. Agitation was predicted by relative preservation in the left dorsal region (*β* = .21; *t*(244) = 3.36; *p* = .001; overall model—*F*(2,244) = 6.14; *p* = .002). Depression was predicted by left dorsal preservation (*β* = .21; *t*(246) = 3.35; *p* = .001; overall model—*F*(2,246) = 7.11; *p* = .001). Irritability was predicted by left dorsal preservation (*β* = .23; *t*(243) = 3.73; *p* < .001; overall model—*F*(2,243) = 6.97; *p* = .001). Models predicting Apathy, Delusions, Aberrant motor behavior, and Sleeping changes were not significant (*p* > .05).

**FIGURE 4:**
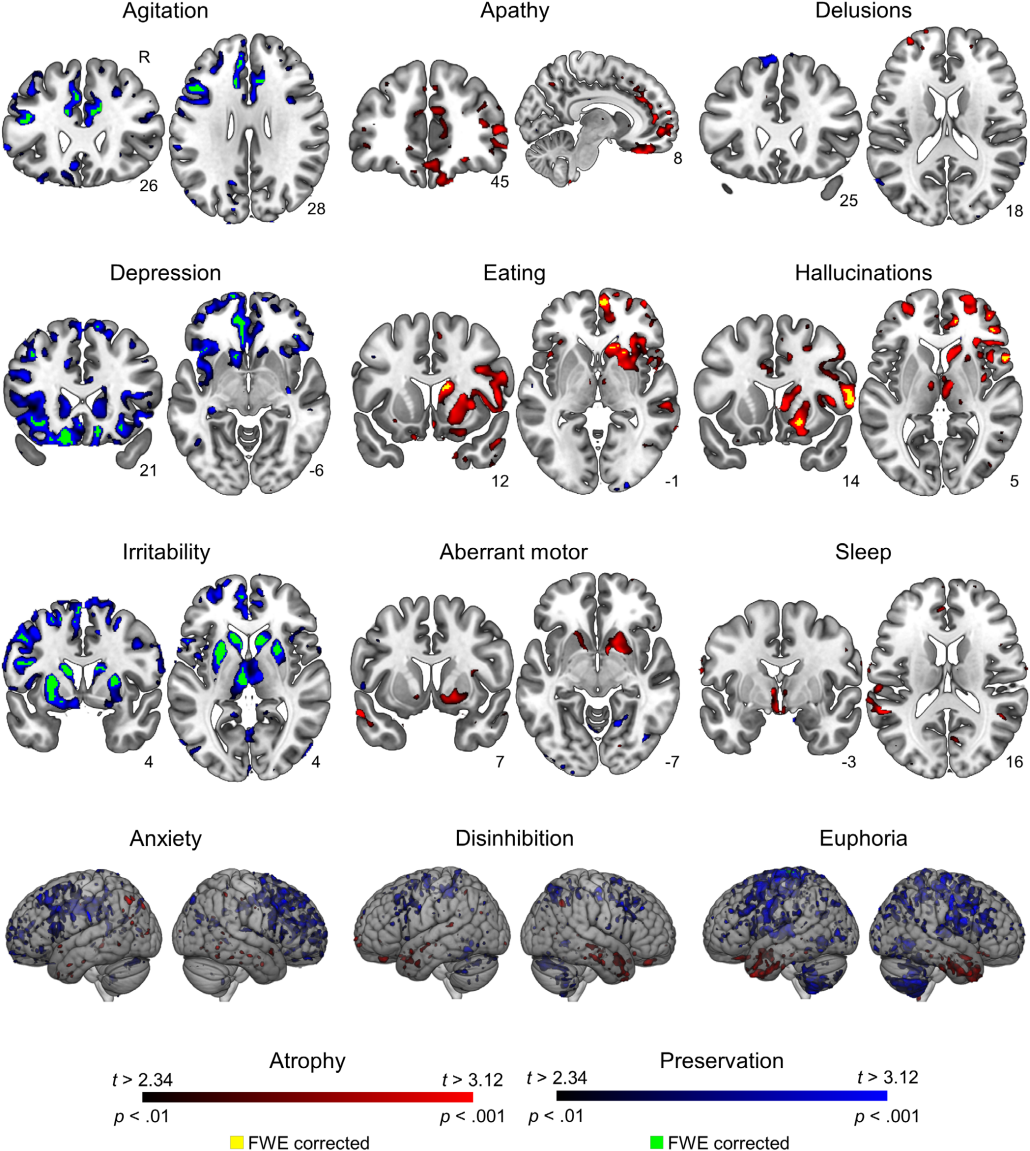
Voxel‐wise brain volume related to neuropsychiatric symptoms in behavioral variant frontotemporal dementia (bvFTD) patients. Atrophy in red color, relative preservation in blue color. Significant areas with peak *p* < .001; FWE < .05 cluster‐corrected displayed in green and yellow.

Voxel‐wise atrophy analysis supported and clarified the results of stepwise regressions (Table 2 and Figure 4). As found by stepwise regression, agitation, depression, and irritability were related to regional relative preservation. Among behaviors associated with DI by clustering and stepwise regression, while anxiety and euphoria were significantly related to preservation of largely dorsal regions, a review of uncorrected maps for these behaviors and disinhibition also suggests an effect of ventral atrophy. On the other hand, the two behaviors associated with AI by clustering, hallucinations and eating behavior, were both related to right‐sided voxel‐wise atrophy.

**TABLE 2:** Voxel‐wise brain volume related to neuropsychiatric symptoms in bvFTD patients.

Brain region(s)		Cluster size (voxels)	Cluster *p*‐value (FWE)	*T*	*x*	*y*	*z*
**Agitation**							
*Preservation*							
Middle and inferior frontal gyri	L	411	.002	4.58	−39	24	24
Paracingulate/anterior cingulate	L	1079	<.001	4.38	−9	33	39
Anterior cingulate cortex	R	337	.008	4.22	9	24	28
**Anxiety**							
*Preservation*							
Middle frontal gyrus	R	402	.004	4.54	36	18	31
**Depression**							
*Preservation*							
Superior frontal gyrus	R	308	.015	5.03	14	39	46
Middle frontal gyrus	L	256	.036	4.53	−37	35	42
Orbitofrontal/subcallosal/Nacc/caudate	L	2477	<.001	4.52	−12	15	−14
Frontal pole	L	444	.002	4.35	−30	63	21
Frontal pole	L	252	.039	4.18	−18	69	4
Orbitofrontal/subcallosal	R	510	.001	4.15	11	33	−20
Orbitofrontal/insula	L	307	.016	4.11	−40	18	−11
Frontal pole/orbitofrontal	R	400	.004	3.9	38	35	−12
**Eating changes**							
*Atrophy*							
Frontal pole	R	836	<.001	4.95	21	53	34
Frontal pole/medial frontal cortex	R	362	.006	4.52	8	56	−5
Caudate/putamen	R	384	.004	3.56	9	8	16
**Euphoria**							
*Preservation*							
Precentral/supplementary motor area	L	1842	<.001	5.72	−23	−12	64
Middle frontal gyrus	L	277	.025	4.56	−40	8	54
Cerebellum	R	268	.029	4.5	23	−52	−51
**Hallucinations**							
*Atrophy*							
Inferior frontal gyrus	R	261	.035	5.35	57	14	6
Superior frontal gyrus	R	290	.021	4.89	20	30	43
Frontal pole	R	326	.012	4.77	32	59	12
Orbitofrontal/putamen	R	240	.049	4.49	18	15	−14
Anterior cingulate/paracingulate	R	404	.004	4.21	8	33	31
Frontal pole	R	273	.028	4.05	17	65	−14
**Irritability**							
*Preservation*							
Putamen/caudate	L	2352	<.001	5.43	−25	6	9
Postcentral	L	661	<.001	5.13	−9	−37	55
Supplementary motor area/superior frontal gyrus	R	2507	<.001	5.05	6	−12	64
Inferior frontal gyrus/precentral	L	840	<.001	4.93	−55	14	34
Precentral	L	401	.003	4.77	−26	−12	64
Superior frontal gyrus	L	395	.004	4.71	−26	9	63
Putamen	R	358	.006	4.55	24	2	9
Orbitofrontal cortex	L	328	.01	4.43	−34	38	−18
Precentral	L	297	.017	4.38	−61	−1	43
Thalamus	L	779	<.001	4.19	−6	−18	16
Superior frontal gyrus/frontal pole	L	310	.014	4.14	−19	36	43
Caudate	R	439	.002	3.99	9	3	18

Abbreviations: bvFTD, behavioral variant frontotemporal dementia; L, left; Nacc, nucleus accumbens; R, right.

### 3.6 Additional investigations of asymmetry and dorsality

**FIGURE 5:**
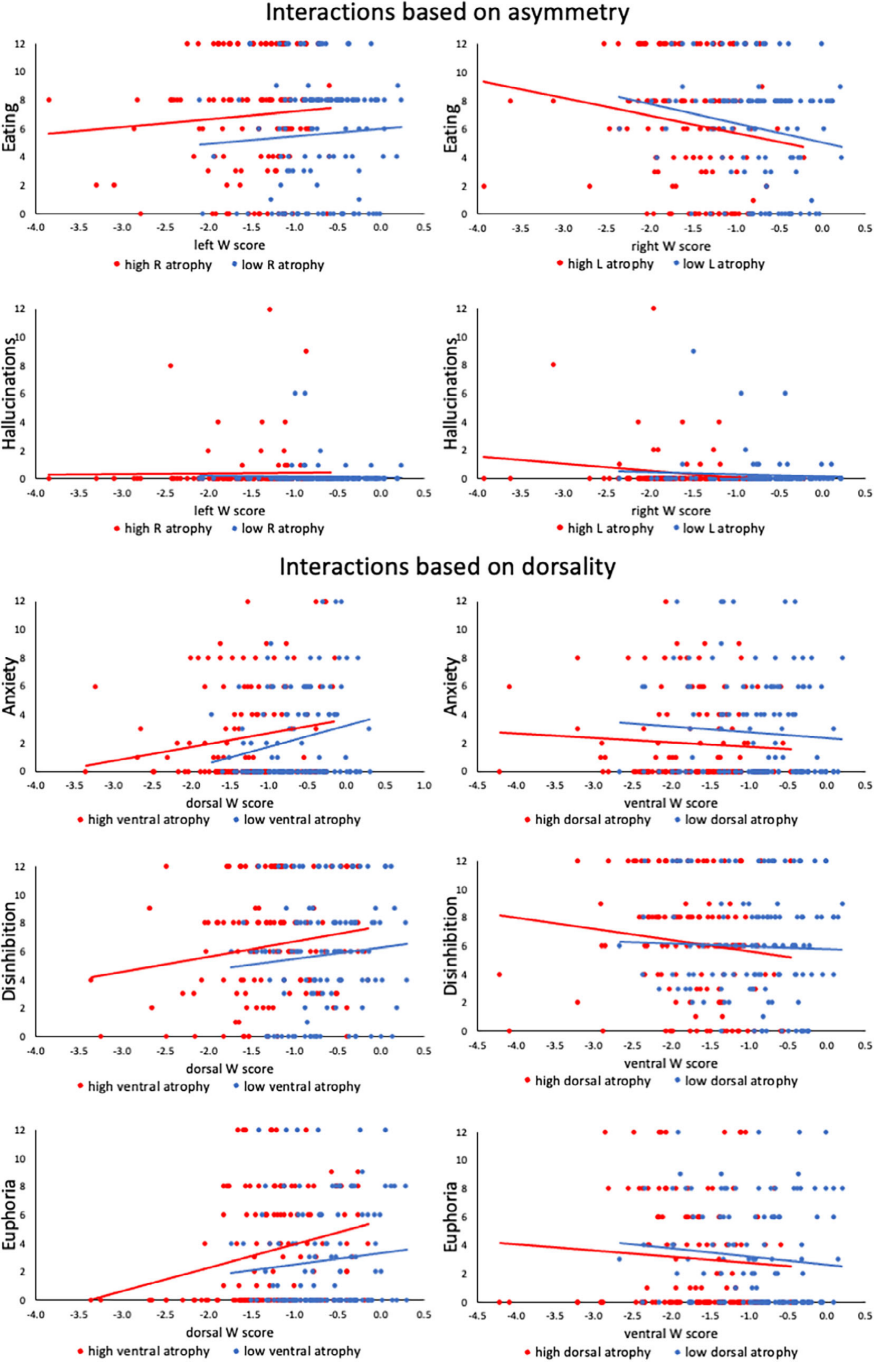
Neuropsychiatric symptom severity and regional volume among behaviors associated with asymmetry or dorsality. The sample was divided according to the median right, left, dorsal, and ventral W‐scores for illustrative purposes to visualize potential interactions between right and left frontotemporal atrophy (eating changes, hallucinations) or the interaction between dorsal and ventral frontotemporal atrophy (anxiety, disinhibition, euphoria).

We further investigated neuropsychiatric symptoms that significantly differed between AI and DI clusters to clarify the relationship between hemispheric or dorsal/ventral regions that best explains the significance of the AI or DI finding. Multiple regression models were performed in the entire sample to examine if significant interaction effects (left × right volume or dorsal × ventral volume) contributed to predicting behavior. Changes in eating behavior were predicted by right frontotemporal atrophy (*β* = −.35; *t*(240) = −2.92; *p* = .004; overall model—*F*(4,240) = 2.98; *p* = .02) but not a left by right interaction (*p* > .05). Hallucinations were not predicted by right and left atrophy or their interaction (*p* > .05). Anxiety was predicted by dorsal preservation (*β* = .39; *t*(244) = 3.09; *p* = .002; overall model—*F*(4,244) = 3.19; *p* = .014). Euphoria was predicted by ventral atrophy (*β* = −.35; *t*(242) = −3.1; *p* = .002) and dorsal preservation (*β* = .25; *t*(242) = 2.02; *p* = .045; overall model—*F*(4,242) = 4; *p* = .004). Disinhibition was predicted by ventral atrophy (*β* = −.30; *t*(240) = 2.65; *p* = .009; overall model—*F*(4,240) = 2.66; *p* = .033). Anxiety, disinhibition, and euphoria were not predicted by a dorsal and ventral atrophy interaction (*p* > .05). Interactions are plotted in Figure 5.

**FIGURE 6:**
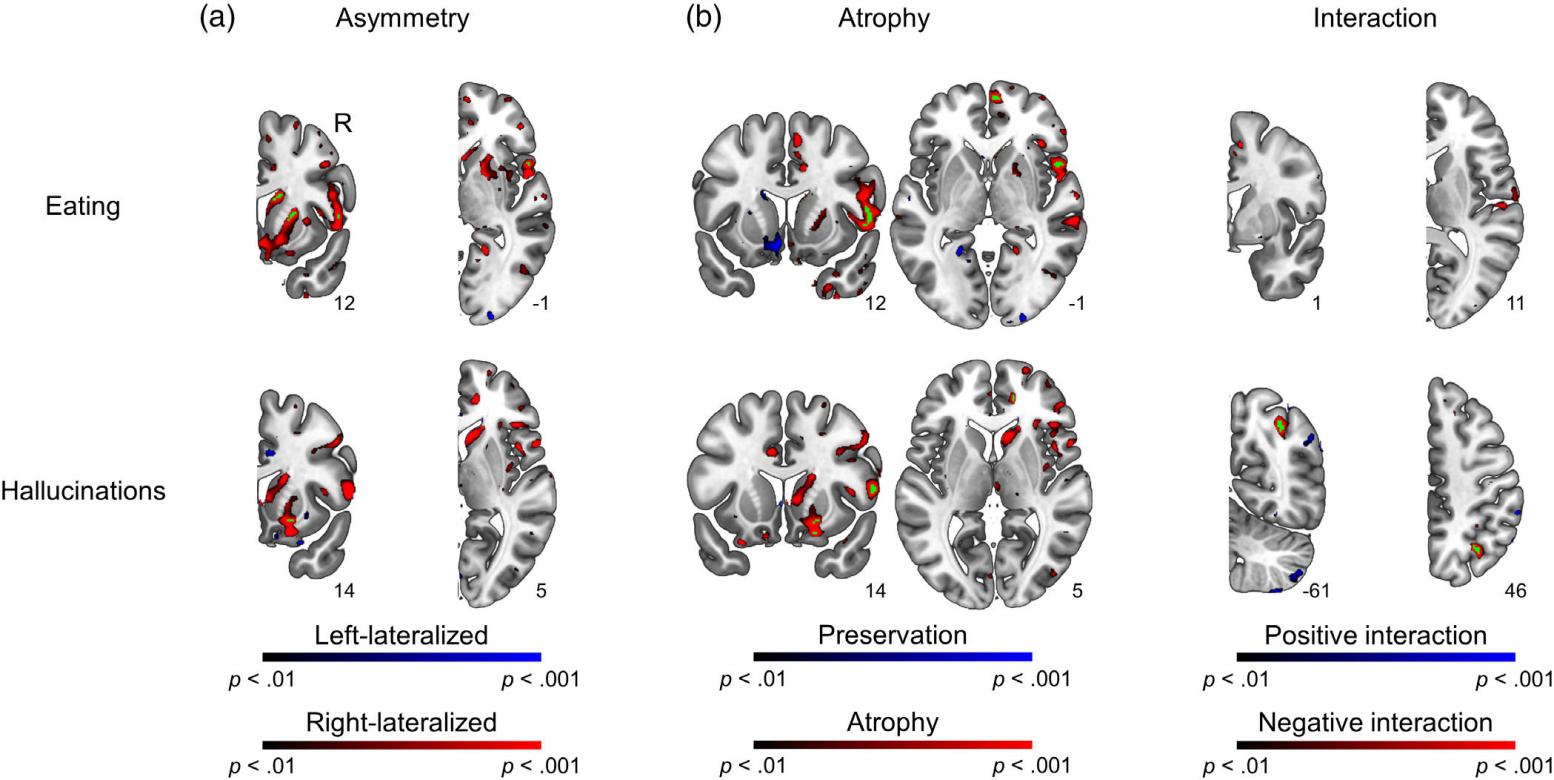
Additional voxel‐wise analyses for eating behavior (top) and hallucinations (bottom). (a) Asymmetry model; (b) ipsilateral atrophy controlling for contralateral atrophy and an ipsilateral × contralateral interaction (left); Interaction model controlling for ipsilateral and contralateral atrophy (right). *p* < .001; FWE < .05 corrected clusters in green.

Voxel‐wise asymmetry analysis revealed regions of right‐lateralized atrophy related to Eating behavior and Hallucinations (Supplementary Table 6 and Figure 6a). Eating behavior was associated with right‐lateralized atrophy in inferior frontal gyrus and basal ganglia, extending into frontal operculum and dorsal insula. Hallucinations were related to basal ganglia as well as both dorsal and ventral right‐lateralized frontal atrophy.

Analysis of the ipsilateral and contralateral interaction revealed a significant positive interaction effect in the right cerebellum (*p* < .001; FWE < .001; *k* = 203; *t* = 4.43; MNI [42 −67 −48]) and a negative interaction effect in the right superior lateral occipital cortex (*p* < .001; FWE = .005; k = 92; *t* = 6.05; MNI [32–60 46]) for hallucinations. Ipsilateral atrophy maps controlled for contralateral atrophy and their interaction revealed a similar pattern of atrophy for both behaviors, although findings were less robust than the model with atrophy alone. There was a cluster of left subcortical preservation related to eating behavior (uncorrected; see Figure 6b).

## 4 DISCUSSION

Heterogeneity in the degree and distribution of bvFTD atrophy has important implications for understanding the phenotypic differences between patients and the mechanisms underlying bvFTD symptoms. This study clarifies how frontotemporal atrophy, especially its asymmetry and dorsality, influences the severity of neuropsychiatric symptoms in patients with bvFTD. Most patients in the study exhibited symmetric atrophy, with approximately equal numbers displaying either a right‐ or left‐asymmetric pattern. While many patients had balanced dorsal and ventral frontotemporal degeneration, substantial imbalance (more than 0.5 SD) was much more commonly ventral than dorsal predominant, in line with previous studies that have reported the most atrophied regions in bvFTD (Ranasinghe et al., 2016; Seeley et al., 2008). There is substantial overlap in atrophy of certain regions among patients with the bvFTD syndrome, reflecting the link between degeneration of these regions and development of the core syndromic features. This makes the degree of overlap of atrophied regions between dorsal and ventral groups, all of whom have bvFTD, expected. There are also unique, non‐overlapping volumetric differences between the clusters. Analyses indicated that individual behavioral symptoms in patients with bvFTD can be related to the degree of asymmetry (abnormal eating behavior); dorsality (euphoria, disinhibition, and anxiety); relative preservation of volume (agitation, irritability, and depression); or atrophy (hallucinations, apathy, and aberrant motor behaviors).

Clustering analyses indicated substantial clinical overlap but also differences in presentation depending on the degree of asymmetry or dorsality. Patients with right‐sided asymmetry had more severe eating behavior changes and hallucinations. Patients with left‐sided asymmetry did worse on several cognitive tests, including language measures. As expected, patients with dorsal predominant atrophy did worse on traditional measures of executive function (Yuan & Raz, 2014) and patients with ventral asymmetry had greater severity on behavioral measures including disinhibition, euphoria, and anxiety.

Analyses of asymmetry and dorsality among genetic and pathological subtypes largely recapitulated prior studies. Patients with *MAPT* mutations have been found to have symmetric atrophy with extensive temporal lobe involvement (Whitwell, Jack, et al., 2009), in keeping with our finding of ventral predominance of atrophy. Although not statistically significant, the majority of patients with *GRN* mutations displayed asymmetry, consistent with prior findings (Rohrer et al., 2010). Clinicopathological studies have shown that certain pathological diagnoses, such as CBD and TDP‐A result in a more dorsal pattern of atrophy, whereas others such as FTLD‐FUS (aFTLD‐U) and Pick's disease tend to be more ventral (Perry et al., 2017). Clusters with balanced right–left and dorsal–ventral atrophy had less atrophy than those that were more imbalanced, potentially reflecting genetic or clinicopathological differences (e.g., enrichment of patients with TDP‐B, including some with *C9orf72* mutations).

### 4.1 Asymmetry and behavior

For eating behavior and hallucinations, the two symptoms associated with asymmetry by clustering, additional analysis helped clarify to what extent behavioral severity related solely to right hemisphere atrophy or to an imbalance between the volume of both right and left. For eating behavior, while voxel‐wise analyses revealed significant findings linking symptom severity to right hemisphere atrophy, and there was no significant left × right interaction, dorsal AI was a stronger predictor of behavioral symptom severity than atrophy. A review of interaction plots suggests that in addition to a negative correlation with right frontotemporal volume, there was a positive correlation between left frontotemporal volume and more severe changes in eating behavior. These analyses suggest that while right hemisphere atrophy has a greater effect on eating behavior, the severity of these symptoms is also greater when there is less atrophy on the left. The association between eating behavior with right‐lateralization fits our hypothesis. A link between degeneration of right frontal cortical and subcortical regions with eating behavior is in line with previous studies that showed overeating in bvFTD being related to right hemisphere atrophy (Perry et al., 2014; Whitwell et al., 2007; Woolley et al., 2007). The involvement of caudate and putamen in this and prior studies is consistent with the role of the striatum in processing rewards. Disruption of regions involved in reward may lead to overeating, craving sweet foods, and increased pursuit of other primary rewards (Perry et al., 2014). The right hemisphere also plays important role in human gustation, including evaluating the affective value of a pleasant taste (Small, 2006).

The association of eating behavior with right‐lateralized asymmetry supports the idea that right hemisphere degeneration may be linked to lower avoidance behavior, with less control of approach behaviors mediated by relative preservation of the left hemisphere (Perry et al., 2014). Hemispheric reward asymmetry is related to approach‐avoidance learning. The left ventral striatum is involved in improved approach learning, whereas the right ventral striatum is linked to avoidance learning (Aberg et al., 2015). Hence, right‐lateralization may lead to increased reward‐seeking behavior related to more intact encoding of positive rewards.

Although initial cluster analysis indicated that hallucinations were also related to right‐lateralization, further analyses showed that this symptom was not related to imbalance of right and left volume, but rather to atrophy in the right hemisphere alone. The presence of psychotic symptoms in FTLD has previously been associated with right‐sided atrophy (Landqvist Waldö et al., 2015), but their localization has not been consistent across studies (Devenney et al., 2017; Devenney et al., 2021). While hallucinations in individuals with FTLD are not rare, and occur more frequently in certain FTLD subtypes (e.g., *C9orf72* carriers), they are less common than in Lewy body disease or Alzheimer's disease (Naasan et al., 2021). A hypothetical framework for hallucinations has been described in a neurodegenerative disease indicating the role of ventral and dorsal attention networks in visual hallucinations (Shine et al., 2015). Our results are in line with their findings considering that attention networks are often more right‐lateralized (Vossel et al., 2014). The low NPI hallucination scores for most patients in this sample may have influenced the findings.

### 4.2 Dorsality and behavior

We found that socioemotional symptoms such as anxiety, euphoria, and disinhibition were related to dorsality, with symptom severity correlating with independent, non‐interacting effects of relative dorsal preservation as well as ventral atrophy. These three symptoms have been previously linked to ventral atrophy. Anxiety is related to medial temporal atrophy (Mah et al., 2015), euphoria and disinhibition have been associated with greater atrophy in the ventromedial prefrontal cortex (Lu et al., 2013), and disinhibition with ventral, more right‐lateralized atrophy (Sheelakumari et al., 2020; Zamboni et al., 2008). Similar behavioral profiles can be observed in certain patients with semantic variant primary progressive aphasia, an FTD variant characterized by ventral atrophy including temporal lobe degeneration, often with preservation of dorsal frontal regions (Ding et al., 2020; Rosen et al., 2006).

While this study focuses on volumetric measurements, dorsal/ventral or right/left structural imbalances may affect behavior through alterations in functional connectivity. Brain atrophy can lead to decreases or even increases in connectivity within larger networks. For example, patients with bvFTD are characterized by decreased salience network connectivity but increased default mode network connectivity (Zhou et al., 2010). Farb et al. (2013) linked reduced frontolimbic connectivity in bvFTD with disinhibition. They also found heightened prefrontal connectivity related to symptom severity. Connectivity between dorsolateral prefrontal cortex and anterior insula was reduced which may impact behavior and affective processes (Farb et al., 2013). Questions regarding the complex relationship between structural and functional changes could not be addressed in the current study.

### 4.3 Regional atrophy or preservation and behavior

While studies of brain–behavior relationships in neurodegenerative disease have often revealed correlations between greater atrophy and greater severity of behavioral symptoms, several behaviors in this study, including agitation, irritability, and depression showed greater severity with regional preservation of volume. The degree of agitation correlated with relative preservation in bilateral anterior cingulate cortex and left dorsolateral prefrontal cortex. Irritability was related to preservation in diffuse dorsal frontal and subcortical regions. Depression was characterized by preservation in dorsal and ventral frontal regions extending to the left insula and caudate. These results are partially in line with previous studies that associated gray matter volume in these brain regions with symptom severity (Bruen et al., 2008; Sellami et al., 2018; Trzepacz et al., 2013). Basavaraju et al. (2022) reported that the presence of depression is related to the preservation of orbitofrontal cortex and anterior cingulate cortical thickness. To interpret cortical preservation we need to consider two points of context. First, relative preservation in this study reflects less severe atrophy than other patients with bvFTD, not preservation relative to volume in healthy controls. Second, regional preservation in this cohort is not present in isolation; rather it coincides with the core cingulate, insula, striatum, and amygdala atrophy that characterizes most patients with bvFTD (Perry et al., 2017). Given that most patients have at least some degree of atrophy in these core bvFTD regions, one interpretation of our results may be that both core bvFTD atrophy and relative preservation elsewhere contribute to the severity of certain symptoms. For example, agitation and irritability are more likely to occur with atrophy in core bvFTD regions and preservation in dorsal ones. Moreover, we should also consider that symptoms are not present in isolation but often co‐occur forming distinct behavioral phenotypes. Different behavioral profiles in bvFTD may have discrete neural correlates (O'Connor et al., 2017; Schroeter et al., 2014). Hence, we cannot rule out the possibility that a single symptom might not be fully explained by the presence of cortical preservation but rather be associated with a certain behavioral phenotype within bvFTD.

Aberrant motor behavior and apathy were not related to asymmetry, dorsality, or preservation in this study, and voxel‐wise atrophy correlations did not persist after correction for multiple comparisons. In the prior literature, these behaviors have been associated with focal atrophy. Striatum atrophy has been previously linked to aberrant motor behavior (Halabi et al., 2013; Josephs et al., 2008; Perry et al., 2012). The striatum is a crucial part of a circuit underlying presence of obsessions and compulsions (Menzies et al., 2008). Apathy has been associated previously with atrophy of regions including right‐sided orbitofrontal cortex, ventromedial prefrontal cortex, anterior cingulate cortex, and dorsolateral prefrontal cortex (Sheelakumari et al., 2020; Zamboni et al., 2008). The anterior cingulate cortex integrates cognitive, emotional, and motivational processes required for effective self‐regulation (Bush et al., 2000; Posner et al., 2007). Its atrophy may lead to apathy in the form of a lack of motivation and alterations in goal‐oriented behavior (Starkstein & Brockman, 2018). A recent review supports the role of the anterior cingulate cortex and medial orbitofrontal cortex in apathetic symptoms (Le Heron et al., 2019).

Finally, delusions and sleep behavior were not clearly explained by either atrophy or preservation. Small, focal regions of atrophy and relative preservation at the uncorrected level preclude definite classification. The absence of significant findings for delusions could relate to low scores across these patients or weak relationships with gray matter volume, and the lack of focal correlates for sleep could relate to the heterogeneity of behaviors and causes that are captured by this NPI domain.

Our study has certain limitations. The use of an informant‐based measure such as NPI, which captures a heterogeneous range of behaviors, may yield different results than would be found with a more specific behavioral test. For example, different forms of apathy or specific modalities of hallucinations may have unique anatomic correlates that would not be identified by the use of NPI scores. The results concerning hallucinations should be viewed with caution as NPI does not distinguish between different types of hallucinations and may not be sensitive to low symptom severity. Our study focused on frontotemporal brain regions, whereas there may be correlates for these behaviors outside of those areas. This study also included only volumetric measures, rather than structural or functional connectivity.

In conclusion, this study provides a conceptual framework for understanding the clinical and anatomic heterogeneity of bvFTD. Frontotemporal atrophy is more often ventral than dorsal, and, while asymmetry is not uncommon, atrophy is most often symmetric, with left‐lateralized atrophy occurring as often as right. The severity of neuropsychiatric symptoms is related to the distribution of atrophy (or its absence) along both right–left and dorsal–ventral axes. Future studies are needed to investigate how behavioral symptoms relate to structural and functional changes to help clarify underlying cognitive component processes. The knowledge gained may suggest new avenues for targeted symptomatic therapy.

## FUNDING INFORMATION

This study was supported by grants P01AG019724 (BLM), P30AG062422 (BLM), R01AG019724 (DCP), and R01AG062758 (DCP) from the National Institutes of Health.

## CONFLICT OF INTEREST STATEMENT

The authors report no competing interests.

## IRB STATEMENT

Written informed consent was obtained from patients or surrogates according to procedures approved by the UCSF Committee on Human Research. This research was conducted with adherence to the Declaration of Helsinki in regards to accepted practices for experimental research with human subjects, and all participants were treated in accordance with the Human Research Protection Program at the University of California, San Francisco.

## Supporting information


**DATA S1** Supporting Information.

## Data Availability

Data are available from the corresponding author upon reasonable request following UCSF Memory and Aging Center procedures (http://memory.ucsf.edu/resources/data).
