# Supplementary material for: Neuropsychiatric symptoms and imbalance of atrophy in behavioral variant frontotemporal dementia
Source: Hum Brain Mapp. 2023 Jul 20;44(15):5013–29. doi: 10.1002/hbm.26428 (PMC10502637; doi:10.1002/hbm.26428)
Supplement: Supplementary file 1 — DATA S1 Supporting Information. [file HBM-44-5013-s001.pdf]

Supplementary material

Supplementary Table 1

Frequency of genetic mutations by asymmetry and dorsality clusters

		MAPT	*FUS*	*TARDBP*	*GRN*	*PSEN1*	*PSEN2*	*APP*	*C9orf72*
All cases	n tested	235	232	231	234	232	232	232	240
	positive	12	0	2	14	0	0	0	44
	% pos of those tested	5.1	0	0.9	6	0	0	0	18.3
	% pos within cohort (n=250)	4.8	0	0.8	5.6	0	0	0	17.6
AI-right	n tested	43	43	43	43	43	43	43	45
	positive	0	0	1	3	0	0	0	0
	% pos of those tested	0	0	2.3	7	0	0	0	0
	% pos within cohort (n=48)	0	0	2.1	6.3	0	0	0	0
AI-symmetric	n tested	137	136	136	136	136	136	136	137
	positive	9	0	1	5	0	0	0	40
	% pos of those tested	6.6	0	0.7	3.7	0	0	0	29.2
	% pos within cohort (n=142)	6.3	0	0.7	3.5	0	0	0	28.2
AI-left	n tested	55	53	52	55	53	53	53	58
	positive	3	0	0	6	0	0	0	4
	% pos of those tested	5.5	0	0	10.9	0	0	0	6.9
	% pos within cohort (n=60)	5	0	0	10	0	0	0	6.7
AI χ^2									χ^2 (2, N = 240) = 25.97; $p < .001$
AI difference									AI-right, AI-left < AI-symmetric
DI-dorsal	n tested	70	69	69	70	69	69	69	72
	positive	0	0	0	8	0	0	0	10
	% pos of those tested	0	0	0	11.4	0	0	0	13.9

	% pos within cohort (n=76)	0	0	0	10.5	0	0	0	13.2
DI-intermediate	n tested	104	103	102	104	103	103	103	106
	positive	3	0	0	5	0	0	0	33
	% pos of those tested	2.9	0	0	4.8	0	0	0	31.1
	% pos within cohort (n=110)	2.7	0	0	4.5	0	0	0	30
DI-ventral	n tested	61	60	60	60	60	60	60	62
	positive	9	0	2	1	0	0	0	1
	% pos of those tested	14.8	0	3.3	1.7	0	0	0	1.6
	% pos within cohort (n=64)	14.1	0	3.1	1.6	0	0	0	1.6
DI χ^2		χ^2 (2, N = 235) = 16.54; $p < .001$							χ^2 (2, N = 240) = 24.12; $p < .001$
Significant posthoc comparisons		DI-dorsal, DI-intermediate < DI-ventral							DI-dorsal, DI-ventral < DI-intermediate

AI, asymmetry index; DI, dorsality index.

Supplementary Table 2

Neuropsychiatric symptom severity by gene mutation status

		Ag	Anx	Apth	Del	Dep	Dis	Eat	Eup	Hal	Irr	Mot	Sle
Mutation negative	n neg	159	160	157	161	161	157	158	159	161	158	158	158
	NPI neg	2.91 (3.40)	2.32 (3.25)	8.25 (3.54)	1.01 (2.53)	1.47 (2.60)	6.19 (3.80)	6.97 (3.89)	3.43 (3.75)	0.31 (1.49)	3.18 (4.12)	6.51 (4.41)	3.29 (3.88)
MAPT	n pos	12	12	12	12	12	12	12	12	12	12	12	12
	NPI pos	2.5833 (3.40)	3.25 (4.47)	6.8333 (4.63)	1.5 (3)	1.9167 (3.73)	7.33 (3.94)	5.33 (3.20)	5.25 (4.77)	0 (0)	2.25 (3.91)	6.5 (3.80)	2 (3.49)
	n neg	221	222	216	223	222	218	218	220	223	219	219	219
	NPI neg	2.88 (3.39)	2.23 (3.15)	8.06 (3.55)	1.03 (2.43)	1.41 (2.55)	6.07 (3.80)	6.43 (4.04)	3.16 (3.65)	0.32 (1.36)	3.07 (3.96)	6.19 (4.38)	2.97 (3.75)
FUS	n pos	0	0	0	0	0	0	0	0	0	0	0	0
	NPI pos	-	-	-	-	-	-	-	-	-	-	-	-
	n neg	230	231	225	232	231	227	227	229	232	228	228	228
	NPI neg	2.88 (3.40)	2.26 (3.18)	8.00 (3.61)	1.03 (2.45)	1.41 (2.53)	6.11 (3.82)	6.37 (4.02)	3.25 (3.74)	0.31 (1.34)	3.07 (3.96)	6.20 (4.33)	2.91 (3.74)
TARDBP	n pos	2	2	2	2	2	2	2	2	2	2	2	2
	NPI pos	1 (1.41)	0 (0)	6 (2.83)	0.5 (0.71)	0 (0)	9 (4.24)	6 (2.83)	5 (1.41)	0 (0)	7 (1.41)	5.5 (3.54)	3 (4.24)
	n neg	227	228	223	229	228	224	224	226	229	225	225	225
	NPI neg	2.90 (3.42)	2.28 (3.19)	8.02 (3.62)	1.04 (2.46)	1.43 (2.55)	6.07 (3.82)	6.37 (4.05)	3.23 (3.75)	0.31 (1.35)	3.01 (3.96)	6.20 (4.35)	2.92 (3.75)
GRN	n pos	14	14	14	14	14	14	13	14	14	14	14	14
	NPI pos	3.14 (3.63)	2.43 (2.95)	8 (3.33)	1.29 (1.68)	1.79 (2.61)	6.36 (3.43)	5.69 (4.39)	3.57 (3.55)	0.86 (1.46)	2.07 (2.89)	4.71 (4.55)	3 (3.80)
	n neg	218	219	213	220	219	215	216	217	220	216	216	216
	NPI neg	2.84 (3.39)	2.23 (3.19)	7.98 (3.63)	1.01 (2.48)	1.37 (2.53)	6.10 (3.83)	6.40 (4.00)	3.23 (3.74)	0.27 (1.32)	3.11 (4.01)	6.27 (4.31)	2.89 (3.73)
PSEN1	n pos	0	0	0	0	0	0	0	0	0	0	0	0
	NPI pos	-	-	-	-	-	-	-	-	-	-	-	-
	n neg	230	231	225	232	231	227	227	229	232	228	228	228
	NPI neg	2.88 (3.40)	2.26 (3.18)	8.00 (3.61)	1.03 (2.45)	1.41 (2.53)	6.11 (3.82)	6.37 (4.02)	3.25 (3.74)	0.31 (1.34)	3.07 (3.96)	6.20 (4.33)	2.91 (3.74)
PSEN2	n pos	0	0	0	0	0	0	0	0	0	0	0	0

	NPI pos	-	-	-	-	-	-	-	-	-	-	-	-
	n neg	230	231	225	232	231	227	227	229	232	228	228	228
	NPI neg	2.88 (3.40)	2.26 (3.18)	8.00 (3.61)	1.03 (2.45)	1.41 (2.53)	6.11 (3.82)	6.37 (4.02)	3.25 (3.74)	0.31 (1.34)	3.07 (3.96)	6.20 (4.33)	2.91 (3.74)
APP	n pos	0	0	0	0	0	0	0	0	0	0	0	0
	NPI pos	-	-	-	-	-	-	-	-	-	-	-	-
	n neg	230	231	225	232	231	227	227	229	232	228	228	228
	NPI neg	2.88 (3.40)	2.26 (3.18)	8.00 (3.61)	1.03 (2.45)	1.41 (2.53)	6.11 (3.82)	6.37 (4.02)	3.25 (3.74)	0.31 (1.34)	3.07 (3.96)	6.20 (4.33)	2.91 (3.74)
C9orf72	n pos	44	44	42	44	43	43	43	43	44	43	43	43
	NPI pos	2.86 (3.59)	2.09 (3.01)	7.64 (3.74)	1.07 (2.38)	1.21 (2.47)	5.19 (4.04)	4.67 (4.17)	1.88 (3.21)	0.20 (0.73)	2.93 (3.65)	5.37 (4.33)	1.81 (3.12)
	n neg	194	195	191	196	196	192	192	194	196	193	193	193
	NPI neg	2.85 (3.35)	2.39 (3.32)	8.09 (3.60)	1.04 (2.45)	1.45 (2.63)	6.32 (3.78)	6.66 (3.91)	3.52 (3.76)	0.32 (1.41)	3.09 (4.01)	6.39 (4.34)	3.09 (3.81)
ANCOVA (significant differences)								$F(3,221) = 4.09$; $p = .007$	$F(3,223) = 3.24$; $p = .023$				
Posthoc comparisons								Negative > *C9orf72*	*MAPT* > *C9orf72*				

Groups with at least four patients were entered into ANOVA (i.e., *C9orf72*, *GRN*, *MAPT*, and mutation negative). Neuropsychiatric Inventory scores reported in the table. Means are reported with standard deviations in the brackets. Ag, Agitation; Anx, Anxiety; Apth, Apathy; Del, Delusions; Dep, Depression; Dis, Disinhibition; Eat; Eating behavior; Eup; Euphoria; Hal, Hallucinations; Irr, Irritability; Mot; Aberrant Motor Behavior; NPI; Neuropsychiatric Inventory; Sle; Sleep changes.

Supplementary Table 3

Frequency of neuropathologic diagnosis by asymmetry clusters

	All cases			Asymmetry Index clusters									Asymmetry Index χ^2	Posthoc comparisons
				right-lateralized			symmetric			left-lateralized				
	N	% of those autopsied (n=95)	% within cohort (n=250)	n	% of those autopsied (n=22)	% within cohort (n=48)	n	% of those autopsied (n=52)	% within cohort (n=142)	n	% of those autopsied (n=21)	% within cohort (n=60)		
Pick's	16	10.3	6.4	15	68.2	31.3	0	0	0	1	4.8	1.7	χ^2 (2, N = 95) = 54.12; p < .001	left-lateralized, symmetric < right-lateralized
CBD	10	6.5	4	1	4.5	2.1	4	7.7	2.8	5	23.8	8.3		
PSP	1	0.6	0.4	0	0	0	1	1.9	0.7	0	0	0		
FTLD-MAPT	3	1.9	1.2	0	0	0	1	1.9	0.7	2	9.5	3.3		
AGD	2	1.3	0.8	0	0	0	2	3.8	1.4	0	0	0		
Unclassifiable-Tau	2	1.3	0.8	1	4.5	2.1	1	1.9	0.7	0	0	0		
Unclassifiable MND-FUS	1	6.5	0.4	0	0	0	1	1.9	0.7	0	0	0		
TDP-A	15	9.7	6	1	4.5	2.1	8	15.4	5.6	6	28.6	0.1		
TDP-B	22	14.2	8.8	0	0	0	18	34.6	12.7	4	19	6.7	χ^2 (2, N = 95) = 10.67; p = .005	right-lateralized < symmetric
TDP-C	4	2.6	1.6	3	13.6	6.3	0	0	0	1	4.8	1.7	χ^2 (2, N= 95) = 7.15; p = .028	symmetric < right-lateralized
TDP-U	10	6.5	4	0	0	0	9	17.3	6.3	1	4.8	1.7		
C9, no TDP	1	0.6	0.4	0	0	0	0	0	0	1	4.8	1.7		
FTLD-FUS (aFTLD-U)	4	2.6	1.6	0	0	0	4	7.7	2.8	0	0	0		
FTLD-UPS	1	0.6	0.4	0	0	0	1	1.9	0.7	0	0	0		
FTLD-ni	1	0.6	0.4	0	0	0	1	1.9	0.7	0	0	0		
indeterminate, non-FTLD	1	0.6	0.4	0	0	0	1	1.9	0.7	0	0	0		
AD	1	0.6	0.4	1	4.5	2.1	0	0	0	0	0	0		

Supplementary Table 4

Frequency of neuropathologic diagnosis by dorsality clusters

	All cases			Dorsality Index clusters									Dorsality Index χ^2	Posthoc comparisons
				dorsal predominant			intermediate			ventral predominant				
	N	% of those autopsied (n=95)	% within cohort (n=250)	n	% of those autopsied (n=37)	% within cohort (n=76)	n	% of those autopsied (n=37)	% within cohort (n=110)	n	% of those autopsied (n=21)	% within cohort (n=64)		
Pick's	16	10.3	6.4	4	10.8	5.3	5	13.5	4.5	7	33.3	10.9		
CBD	10	6.5	4	9	24.3	11.8	1	2.7	0.9	0	0	0	χ^2 (2, N = 95) = 12.36; *p* = .002	ventral, intermediate < dorsal predominant
PSP	1	0.6	0.4	1	2.7	1.3	0	0	0	0	0	0		
FTLD-MAPT	3	1.9	1.2	0	0	0	0	0	0	3	14.3	4.7		
AGD	2	1.3	0.8	0	0	0	2	5.4	1.8	0	0	0		
Unclassifiable-Tau	2	1.3	0.8	1	2.7	1.3	0	0	0	1	4.8	1.6		
Unclassifiable MND-FUS	1	6.5	0.4	1	2.7	1.3	0	0	0	0	0	0		
TDP-A	15	9.7	6	11	29.7	14.5	4	10.8	3.6	0	0	0	χ^2 (2, N = 95) = 10.04; *p* < .001	ventral predominant < dorsal predominant
TDP-B	22	14.2	8.8	7	18.9	9.2	13	35.1	11.8	2	9.5	3.1		
TDP-C	4	2.6	1.6	0	0	0	0	0	0	4	19	6.2	χ^2 (2, N = 95) = 14.72; *p* = .001	dorsal, intermediate < ventral predominant
TDP-U	10	6.5	4	2	5.4	2.6	8	21.6	7.3	0	0	0		
C9, no TDP	1	0.6	0.4	0	0	0	1	2.7	0.9	0	0	0		
FTLD-FUS (aFTLD-U)	4	2.6	1.6	0	0	0	1	2.7	0.9	3	14.3	4.7		
FTLD-UPS	1	0.6	0.4	0	0	0	0	0	0	1	4.8	1.6		
FTLD-ni	1	0.6	0.4	0	0	0	1	2.7	0.9	0	0	0		
indeterminate, non-FTLD	1	0.6	0.4	0	0	0	1	2.7	0.9	0	0	0		
AD	1	0.6	0.4	1	2.7	1.3	0	0	0	0	0	0		

Supplementary Table 5

Neuropsychiatric symptom severity by neuropathologic diagnosis

	N	Ag	Anx	Apth	Del	Dep	Dis	Eat	Eup	Hal	Irr	Mot	Sle
Pick's	16	3 (3.67)	1.06 (2.21)	8.94 (2.86)	1.31 (3.05)	1.13 (3.10)	5.38 (3.30)	8.38 (2.85)	3 (3.16)	0.56 (2.25)	2.88 3.70)	7.25 (5.04)	1.75 (2.74)
CBD	10	1.3 (1.64)	1.3 (2.21)	7.33 (3.84)	0 (0)	1.7 (2.91)	5.56 (2.19)	7.9 (3.84)	1.5 (2.46)	0 (0)	1.8 (2.44)	5 (2.92)	2.8 (3.71)
PSP	1	-	-	-	-	-	-	-	-	-	-	-	-
FTLD-MAPT	3	4.33 (6.66)	2.33 (1.53)	7.33 (4.16)	4 (4.58)	0 (0)	8 (3.46)	5.33 (4.62)	8 (6.93)	0 (0)	5.67 (5.69)	7.33 (1.15)	3.33 (4.93)
AGD	2	4 (0)	2 (2.83)	8 (0)	4 (5.66)	2.5 (2.12)	8 (5.66)	6 (2.83)	7 (1.41)	0 (0)	3 (4.24)	9 (4.24)	4 (5.66)
Unclassifiable-Tau	2	7 (1.41)	0 (0)	8 (0)	0 (0)	4 (5.66)	8 (5.66)	6 (2.83)	8 (0)	0 (0)	4 (2.83)	8 (0)	4 (5.66)
Unclassifiable MND-FUS	1	-	-	-	-	-	-	-	-	-	-	-	-
TDP-A	15	1.6 (3.38)	2 (2.93)	9.14 (3.01)	1.47 (2.45)	1.64 (2.87)	4.93 (4.11)	4.86 (4.47)	1.93 (3.39)	0.73 (1.44)	0.87 (2.23)	4.6 (4.93)	3.43 (3.44)
TDP-B	22	2.59 (3.28)	2.41 (3.76)	8.91 (3.25)	1.68 (3.67)	0.59 (1.82)	5.5 (3.85)	6.23 (4.71)	3.32 (4.28)	0.18 (0.50)	2.77 (3.98)	7.09 (4.30)	3 (3.88)
TDP-C	4	6 (4.90)	4.5 (5.74)	7 (5.03)	0.5 (0.58)	0 (0)	11 (2)	5.5 (3.79)	4.5 (3.11)	0 (0)	6.5 (5)	4.5 (5.74)	0.33 (0.58)
TDP-U	10	2.5 (3.17)	0.4 (1.26)	7.44 (4.50)	1.8 (3.55)	1 (2.16)	4.89 (4.11)	5.89 (3.69)	0.56 (0.73)	0 (0)	1.67 (2.65)	5.89 (4.59)	2.7 (3.71)
C9, no TDP	1	-	-	-	-	-	-	-	-	-	-	-	-
FTLD-FUS (aFTLD-U)	4	7.5 (3)	5 (3.46)	12 (0)	0 (0)	1 (2)	12 (0)	11 (2)	7 (5.03)	0 (0)	4 (5.66)	12 (0)	10 (4)
FTLD-UPS	1	-	-	-	-	-	-	-	-	-	-	-	-
FTLD-ni	1	-	-	-	-	-	-	-	-	-	-	-	-
indeterminate, non-FTLD	1	-	-	-	-	-	-	-	-	-	-	-	-
AD	1	-	-	-	-	-	-	-	-	-	-	-	-
ANCOVA (significant differences)		F(6,73) = 2.72; *p* = .019							F(6,71) = 3.76; *p* = .003				F(6,71) = 3.47; *p* = .005
Posthoc comparisons		aFTLD-U > CBD, TDP-A							aFTLD-U > Pick's, TDP-A, TDP-B, TDP-U				aFTLD-U > CBD, TDP-A, TDP-B, TDP-C, TDP-U

Groups with at least four patients were entered into the analysis (i.e., *aFTLD-U*, *CBD*, *Pick's*, *TDP-A*, *TDP-B*, *TDP-C*, and *TDP-U*). Neuropsychiatric Inventory scores reported in the table. Means are reported with standard deviations in the brackets. Ag, Agitation; Anx, Anxiety; Apth, Apathy; Del, Delusions; Dep, Depression; Dis, Disinhibition; Eat; Eating behavior; Eup; Euphoria; Hal, Hallucinations; Irr, Irritability; Mot; Aberrant Motor Behavior; Sle; Sleep changes.

Supplementary Table 6

Voxel-wise asymmetry analysis

Brain region(s)	Cluster size (voxels)	Cluster p-value (FWE)	peak t	x	y	z
**Eating changes**						
Right-lateralized						
Putamen	178	.004	4.38	20	3	6
IFG	110	.044	4.22	53	12	3
Caudate	200	.002	4.00	15	14	17
**Hallucinations**						
Right-lateralized						
Caudate	199	.002	5.36	15	29	-3
OFC / Putamen	109	.048	4.61	20	12	-18
SFG	176	.005	4.51	14	39	50

IFG, inferior frontal gyrus; OFC, orbitofrontal cortex; SFG, superior frontal gyrus

List of regions of interests (ROIs) in frontotemporal mask. ROIs taken from Brainnetome Atlas (https://atlas.brainnetome.org).

Dorsal regions

**Lobe**	**Gyrus**	**Left and Right Hemisphere**	**Label L**	**Label R**	**Anatomical and modified Cyto-architectonic descriptions**
**Frontal Lobe**	SFG, Superior Frontal Gyrus	SFG_L(R)_7_1	1	2	*A8m, medial area 8*
		SFG_L(R)_7_2	3	4	*A8dl, dorsolateral area 8*
		SFG_L(R)_7_3	5	6	*A9l, lateral area 9*
		SFG_L(R)_7_4	7	8	*A6dl, dorsolateral area 6*
		SFG_L(R)_7_5	9	10	*A6m, medial area 6*
		SFG_L(R)_7_6	11	12	*A9m,medial area 9*
		SFG_L(R)_7_7	13	14	*A10m, medial area 10*
	MFG, Middle Frontal Gyrus	MFG_L(R)_7_1	15	16	*A9/46d, dorsal area 9/46*
		MFG_L(R)_7_2	17	18	*IFJ, inferior frontal junction*

		MFG_L(R)_7_3	19	20	*A46, area 46*
		MFG_L(R)_7_4	21	22	*A9/46v, ventral area 9/46*
		MFG_L(R)_7_5	23	24	*A8vl, ventrolateral area 8*
		MFG_L(R)_7_6	25	26	*A6vl, ventrolateral area 6*
		MFG_L(R)_7_7	27	28	*A10l, lateral area10*
	IFG, Inferior Frontal Gyrus	IFG_L(R)_6_1	29	30	*A44d,dorsal area 44*
		IFG_L(R)_6_2	31	32	*IFS, inferior frontal sulcus*
		IFG_L(R)_6_3	33	34	*A45c, caudal area 45*
		IFG_L(R)_6_4	35	36	*A45r, rostral area 45*
		IFG_L(R)_6_5	37	38	*A44op, opercular area 44*
		IFG_L(R)_6_6	39	40	*A44v, ventral area 44*
**Insular Lobe**	INS, Insular Gyrus	INS_L(R)_6_3	167	168	*dIa, dorsal agranular insula*
		INS_L(R)_6_5	171	172	*dIg, dorsal granular insula*
		INS_L(R)_6_6	173	174	*dId, dorsal dysgranular insula*
**Limbic Lobe**	CG, Cingulate Gyrus	CG_L(R)_7_2	177	178	*A24rv, rostroventral area 24*

		CG_L(R)_7_3	179	180	*A32p, pregenual area 32*
		CG_L(R)_7_5	183	184	*A24cd, caudodorsal area 24*
**Subcortical Nuclei**	BG, Basal Ganglia	BG_L(R)_6_5	227	228	*dCa, dorsal caudate*
		BG_L(R)_6_6	229	230	*dlPu, dorsolateral putamen*

Ventral regions

Lobe	Gyrus	Left and Right Hemisphere	Label L	Label R	Anatomical and modified Cyto-architectonic descriptions
**Frontal Lobe**	OrG, Orbital Gyrus	OrG_L(R)_6_1	41	42	*A14m, medial area 14*
		OrG_L(R)_6_2	43	44	*A12/47o, orbital area 12/47*
		OrG_L(R)_6_3	45	46	*A11l, lateral area 11*
		OrG_L(R)_6_4	47	48	*A11m, medial area 11*
		OrG_L(R)_6_5	49	50	*A13, area 13*
		OrG_L(R)_6_6	51	52	*A12/47l, lateral area 12/47*
**Temporal Lobe**	STG, Superior Temporal Gyrus	STG_L(R)_6_1	69	70	*A38m, medial area 38*
		STG_L(R)_6_3	73	74	*TE1.0 and TE1.2*
		STG_L(R)_6_4	75	76	*A22c, caudal area 22*
		STG_L(R)_6_5	77	78	*A38l, lateral area 38*
		STG_L(R)_6_6	79	80	*A22r, rostral area 22*
	MTG, Middle Temporal Gyrus	MTG_L(R)_4_1	81	82	*A21c, caudal area 21*
		MTG_L(R)_4_2	83	84	*A21r, rostral area 21*

		MTG_L(R)_4_4	87	88	*aSTS, anterior superior temporal sulcus*
	ITG, Inferior Temporal Gyrus	ITG_L(R)_7_1	89	90	*A20iv, intermediate ventral area 20*
		ITG_L(R)_7_3	93	94	*A20r, rostral area 20*
		ITG_L(R)_7_4	95	96	*A20il, intermediate lateral area 20*
		ITG_L(R)_7_7	101	102	*A20cv, caudoventral of area 20*
**Insular Lobe**	INS, Insular Gyrus	INS_L(R)_6_2	165	166	*vIa, ventral agranular insula*
		INS_L(R)_6_4	169	170	*vId/vIg, ventral dysgranular and granular insula*
**Limbic Lobe**	CG, Cingulate Gyrus	CG_L(R)_7_7	187	188	*A32sg, subgenual area 32*
**Subcortical Nuclei**	Amyg, Amygdala	Amyg_L(R)_2_1	211	212	*mAmyg, medial amygdala*
		Amyg_L(R)_2_2	213	214	*lAmyg, lateral amygdala*
	BG, Basal Ganglia	BG_L(R)_6_3	223	224	*NAC, nucleus accumbens*
